# Clinical evaluation of efficacy and safety of cyclosporine (Imusporin) in renal transplant patients with stable graft function maintained on neoral or bioral

**DOI:** 10.4103/0970-1591.32062

**Published:** 2007

**Authors:** Mrigank S. Jha, Aneesh Srivastava, Deepak Dubey, Amit Gupta, R. K. Sharma, Anant Kumar

**Affiliations:** Department of Urology and Renal Transplantation, Sanjay Gandhi Post Graduate Institute of Medical Sciences, Lucknow, UP, India; * Department of Nephrology, Sanjay Gandhi Post Graduate Institute of Medical Sciences, Lucknow, UP, India

**Keywords:** Cyclosporine, efficacy, safety

## Abstract

**Objective:**

Previous pharmacokinetic studies have demonstrated bioequivalence of Imusporin (microemulsion preparation of cyclosporine, Cipla) to the innovator product Neoral (Novartis, Switzerland). This study was done to evaluate the clinical efficacy and safety of Imusporin in patients who have already undergone renal transplant and have stable graft function maintained on cyclosporine preparation other than Imusporin.

**Materials and Methods:**

Twenty-two renal allograft recipients (mean age of 31.77 years, range 18-53 years), with stable graft function, previously on Neoral or Bioral were switched over to Imusporin after recording their relevant baseline clinical and biochemical parameters. These were repeated on 1, 4, 7, 15, 30 and 90 days after the start of therapy. Change in dosage required to maintain C2 levels at each visit were analyzed by paired sample *t*-test. Safety of the drug was assessed by the type and severity of adverse events developed during the therapy. Cost analysis was done assuming an average maintenance immunosuppression dose of 150 mg/day of cyclosporine.

**Results:**

Twenty-one patients completed the study. One patient was lost to follow-up. Mean C2 value before switchover was 894 ± 208 ng/ml, which was not significantly different from the mean values of C2 after switchover therapy (*P*>0.30). Change in dosage required to maintain C2 levels was not significantly different from the baseline dose of 2.34 mg/ kg body weight (*P*>0.1). No patient developed graft rejection after switchover therapy at a median follow-up of 16 months (14-18 months). Mean baseline SCr was similar to SCr at day 90 (1.38 vs. 1.37 mg/dl, *P*=0.930). No severe adverse events were reported. Mild side-effects included headache (4), somnolence (2), dry mouth (5) and generalized fatigue (6). Use of Imusporin (Cipla, India) results in an annual savings of Rs. 19892 over Neoral (Novartis, Switzerland) and Rs. 2263 over Bioral (Panacea Biotech, India).

**Conclusions:**

Imusporin is clinically as safe and efficacious as other cyclosporine preparations available while significantly reducing the cost of treatment.

In developing countries like India, the cost of immunosuppression following renal transplantation is a major issue. There has been a constant attempt to evolve native cost-effective alternatives without compromising the safety of the graft.[1]

Cyclosporine has stood the test of time and has emerged as a major pillar for any immunosuppression regime.[2] Neoral (Novartis, Switzerland) is a microemulsion formulation with minimum intrapatient and interpatient pharmacokinetic variability[3] notwithstanding its prohibitive costs as per our economic standards.

Bioral (Panacea Biotech, India) was introduced as an equally efficacious and bioequivalent cyclosporine microemulsion formulation at reduced costs. However, to the best of our knowledge, there is no published clinical study on transplant patients.

The obvious need for an alternative safe and efficacious cyclosporine preparation which is equivalent to the original product, yet cost-effective, cannot be denied. Imusporin (Cipla, India) is a microemulsion formulation of cyclosporine at a lesser cost and could be economically beneficial to our patients in the long run.

Although the bioequivalence and safety of Imusporin has already been established in healthy young volunteers at Dunedin, New Zealand in a study conducted by Cipla, there is enough evidence to show that CsA pharmacokinetics is different in transplant patients treated chronically with CsA.[4] Furthermore, it is well known that there could be inter-racial variations in pharmacokinetics of the drug.

This study was therefore designed on the native stable transplant population to evaluate the safety and efficacy of Imusporin and to assess its cost-effectiveness.

## MATERIALS AND METHODS

Twenty-two renal allograft recipients (mean age of 31.77 years, range 18-53 years), with stable graft function, previously on Neoral or Bioral were switched over to Imusporin after recording their baseline serum creatinine (SCr), cyclosporine levels after two hours of drug administration (C2 levels), dose of cyclosporine, body weight and relevant hematological and biochemical parameters including complete blood counts, renal function tests, liver function tests and blood sugar levels. Subject inclusion criteria were: (a) patients of either sex aged more than 18 years with renal transplantation and stable graft function for at least six months (b) subjects who were able to give informed consent and agreed to regular follow-up.

Subject exclusion criteria were: (a) patients with significant hepatic disease, uncontrolled hypertension/diabetes mellitus (b) patients on concomitant drug therapy which is likely to increase or decrease cyclosporine levels or enhance its nephrotoxicity (c) patients with severe or unstable angina pectoris within the previous one month (d) history of hypersensitivity to the trial medication (e) doubts on patient compliance.

Thirteen patients were on Bioral and nine were on Neoral prior to the start of the study. Mean duration of transplantation at the start of Imusporin was 28.59 months (range 6-53 months). Parameters were repeated on 1, 4, 7, 15, 30 and 90 days after the start of therapy.

The efficacy of Imusporin was assessed by evaluating the following parameters:

(a) Primary efficacy parameter was the change in dosage of cyclosporine (Imusporin) required to maintain C2 levels (b) Secondary efficacy parameters were (i) incidence of graft rejection episodes during the study and (ii) graft function maintenance based on any change in serum creatinine.

Change in dosage required to maintain C2 levels at each visit was analyzed by paired sample t-test.

Safety of the drug was assessed by the type and severity of adverse events developed during the therapy.

Cost analysis was done assuming an average maintenance immunosuppression dose of 150 mg/day of cyclosporine.

## RESULTS

**Figure 1:**
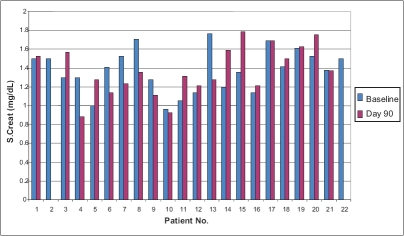
S. Creatinine (Baseline Vs Day 90)

**Figure 2:**
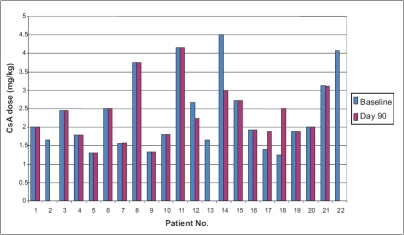
CsA dose (Baseline Vs Day 90)

**Figure 3:**
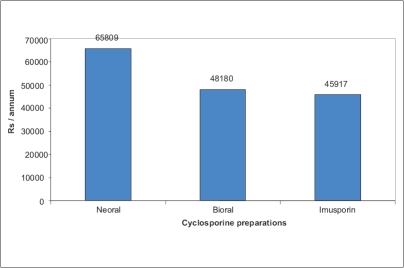
Cost analysis (CsA 150 mg/day)

Twenty-one patients completed the study. One patient was lost to follow-up. Mean C2 value before switchover was 894 ± 208 ng/ml, which was not significantly different from the mean values of C2 after switchover therapy (852, 877, 880, 904, 945, 903 ng/ml on days 1, 4, 7, 15, 30 and 90 respectively, *P*>0.30). Change in dosage required to maintain C2 levels was not significantly different from the baseline dose of 2.34 mg/ kg body weight (*P*>0.1) [Figure 1]. No patient developed graft rejection after switchover therapy at a median follow-up of 16 months (14-18 months). Mean baseline SCr was similar to SCr at day 90 (1.38 vs. 1.37 mg/dl, *P*=0.930) [Figure 2]. No severe adverse events were reported. Mild side-effects included headache (4), somnolence (2), dry mouth (5) and generalized fatigue (6). Use of Imusporin (Cipla, India) resulted in an annual savings of Rs.19892 over Neoral (Novartis, Switzerland) and Rs. 2263 over Bioral (Panacea Biotech, India) [Figure 3].

## DISCUSSION

There could be potential implications of substitution of generic cyclosporine formulations for the original product Neoral due to the narrow therapeutic index of cyclosporine. Whereas Carnahan and Cooper *et al*[5] have reported that Neoral and Gengraf (one of such generics studied at Nashville, Tennessee, USA) were therapeutically equivalent CsA formulations and advocated 1:1 conversion, there are concerns raised by others.[4,6–8]

Based on the available literature, it is clear that acute rejections are more common in the initial six months following transplantation. We therefore included only those patients who had stable graft function following at least six months of renal transplantation, in order to minimize any possible adverse effects on graft function.

Ideally, the study should have included only those patients who were on Neoral, this being the innovator product. However, some patients who were on Bioral were also included as a large proportion of the study population was on this drug due to its lower cost and it being an established product in the country. Further, the patients who were on Neoral were generally more reluctant to switch over to a generic preparation due to inherent fear.

Throughout the study period, we monitored serum creatinine, C2 levels and the incidence of hospitalization for graft rejection or CsA toxicity as primary markers of safety and efficacy of our therapeutic conversion. We also monitored the need for dose adjustments after conversion to Imusporin as a marker for therapeutic equivalence of the two products.

Our data documents no significant differences in any monitored parameters following conversion from Neoral / Bioral to Imusporin. Carnahan *et al* have also reported similar findings in a well-designed study from Tennessee, USA.[5] We also observed that C2 levels remained unchanged after conversion to Imusporin.

As economics was the major driving force behind this study, the cost analysis was done very meticulously and projected as annual savings. It is clear from our data that there could be substantial savings on an annual basis if Imusporin is used and it could translate into mammoth amounts if calculated for a five or 10-year period.

Some limitations of this study include the small sample size which increases the probability of making a type II error; and the short duration of follow-up which limits the ability to assess long-term safety and graft survival data. The study population includes patients only from the northern and central parts of the country and the study subjects were not randomized.

## CONCLUSION

Our results indicate that Neoral / Imusporin and Bioral / Imusporin are therapeutically equivalent CsA formulations and renal transplant patients maintained on Neoral can be safely and effectively switched over to Imusporin based on a 1:1 conversion ratio.

In addition, Neoral to Imusporin conversion presents a substantial cost-saving opportunity.

However, the present study has demonstrated the clinical safety and efficacy of Imusporin in only a limited number of stable renal transplant recipients on maintenance immunosuppression and the results cannot be generalized to the population of recipients for either prevention of rejection (maybe these patients do not need CsA after all) or for initial therapy.
